# Rituximab maintenance significantly reduces early follicular lymphoma progressions in patients treated with frontline R‐CHOP

**DOI:** 10.1002/jha2.60

**Published:** 2020-07-31

**Authors:** Vít Procházka, David Belada, Andrea Janíková, Kateřina Benešová, Heidi Mociková, Juraj Ďuraš, Jan Pirnos, Kateřina Kopečková, Vít Campr, Tomáš Fürst, Robert Pytlík, Alice Sýkorová, Jozef Michalka, Jitka Dlouhá, Tomáš Papajík, Marek Trněný

**Affiliations:** ^1^ Department of Hemato‐Oncology, Faculty of Medicine and Dentistry University Hospital in Olomouc Olomouc Czech Republic; ^2^ Fourth Department of Internal Medicine – Hematology University Hospital Hradec Králové Czech Republic; ^3^ Department of Hematology and Oncology Masaryk University Hospital Brno Brno Czech Republic; ^4^ First Department of Internal Medicine – Hematology First Faculty of Medicine and General Teaching Hospital Prague Czech Republic; ^5^ Department of Internal Medicine and Hematology, Faculty Hospital Kralovske Vinohrady and Third Faculty of Medicine Charles University Prague Czech Republic; ^6^ Department of Hemato‐Oncology University Hospital Ostrava Ostrava Czech Republic; ^7^ Department of Oncology Hospital České Budějovice České Budějovice Czech Republic; ^8^ Department of Oncology University Hospital Motol Prague Czech Republic; ^9^ Department of Pathology and Molecular Medicine University Hospital in Motol Prague Czech Republic; ^10^ Second Faculty of Medicine Charles University Prague Czech Republic; ^11^ Department of Mathematical Analysis and Applications of Mathematics, Faculty of Science Palacký University Olomouc Czech Republic

**Keywords:** early progression, follicular lymphoma, maintenance, rituximab

## Abstract

Twenty percent of patients with high‐tumor‐burden (HTB) follicular lymphoma (FL) develop progression/relapse of disease (POD) within 24 months of frontline immunochemotherapy. Unfortunately, about 50% of these patients die within 5 years since POD event. Rituximab maintenance was proven to reduce relapse rate in responding FL, but its role on preventing POD was not defined. We analyzed 1360 HTB‐FL patients from the Czech Lymphoma Study Group registry treated with frontline rituximab‐containing regimen. Of those, 950 cases received rituximab plus cyclophosphamide, doxorubicin, vincristine, and prednisone (R‐CHOP) and achieved complete or partial remission: 712 patients received rituximab maintenance (MAINT) and 238 were a historical observational cohort (OBS). We have proposed a modified POD24 (mPOD24) endpoint for the chemosensitive patients calculated from the end‐of‐induction (EOI). Survival rates since EOI were as follows: 5‐year overall survival (OS) 86.2% versus 94.5% in the OBS and MAINT groups, respectively (*P* < .001) and 5‐year progression‐free survival 58.5% (OBS) and 75.4% (MAINT) (*P* < .001). The Cox proportional hazards model showed a decrease in mPOD24 incidence in the MAINT group with the overall hazard rate reduced by 56% (hazard ratio = 0.44; *P* < .001). The cumulative incidence of mPOD24 was reduced from 24.1% in OBS to 10.1% in MAINT (*P* < .001). Comparison of non‐mPOD24 cases showed OS similar to that in the general population. Rituximab maintenance given after R‐CHOP resulted in a 2.4‐fold reduction in mPOD24 incidence. Once the non‐POD24 status is achieved, FL does not shorten the patients’ life expectancy.

## 1 INTRODUCTION

Follicular lymphoma (FL) represents the most frequent subtype of indolent B‐cell lymphomas in the United States and Western countries, making up 20‐30% of all non‐Hodgkin lymphoma cases [1]. In Central Europe (the Czech Republic), the incidence is about 19%, as seen from the Czech Lymphoma Study Group (CLSG) registry data [2]. With a median age at diagnosis of 60 years and usually good performance status of the patients, it also represents a malignancy with a broad spectrum of frontline treatment options [3].

The indication for systemic therapy comes along with fulfillment of criteria for “high‐tumor‐burden” disease (HTB) [4]. Standard therapy in HTB‐FL patients is immunochemotherapy, optionally followed by maintenance immunotherapy in responding cases [4, 5]. Although the optimal treatment protocol is still a matter of debate, rituximab with cyclophosphamide, doxorubicin, vincristine, and prednisone (R‐CHOP); rituximab with cyclophosphamide, vincristine, and prednisone (R‐CVP); and bendamustine with rituximab (BR) are the most recommended options [6, 7, 8, 9]. However, frontline therapy in HTB‐FL is still neither risk nor stage adapted.

Despite the improved effectiveness of therapy, about 13‐20% of high‐risk patients still do not benefit from standard rituximab‐based induction [10, 11]. Data from the randomized prospective Primary Rituximab and Maintenance (PRIMA) study showed a poorer outcome in those with high‐risk disease in terms of lower complete response rate, higher risk of relapse, and lower efficacy of maintenance therapy [8].

The natural course of HTB‐FL being treated with systemic therapy was believed to be inevitably relapsing. Subsequent relapses were thought not to shorten the lives of patients (Swenson). This conservative view was changed by a retrospective analysis of data from the National LymphoCare Study that identified early relapse after R‐CHOP as a poor outcome predictor. Patients who progressed or relapsed within 24 months after diagnosis (POD24) were at a high risk for lymphoma‐related death [11]. In this pivotal analysis, POD24 was associated with a 6.44‐times higher risk of death as compared to patients without an early event. The validity of this endpoint was repeatedly and independently confirmed using data from prospective trials [12, 13, 14].

Identification of early progression of disease, or POD24, became a robust surrogate endpoint for defining ultrahigh‐risk FL patients [15]. Indeed, it separated a population with different biology and thus in need of a different treatment approach [16, 17].

The treatment goal in HTB‐FL should be to avoid a POD24 event, with maintenance immunotherapy being a solution for a significant proportion of FL patients who respond well to the initial immunochemotherapy regimen. Frontline response consolidation is still considered optional only and data about the efficacy of rituximab maintenance (RM) in terms of POD24 reduction, after remission is achieved, are missing. For this population, we have proposed “modified POD24” (mPOD24) endpoint calculated from the date of the last chemotherapy and thus covering anticipated length of maintenance application.

We analyzed a large cohort of unselected, consecutively enrolled FL patients from the Czech Republic who were treated with the same rituximab‐containing induction regimen (R‐CHOP) and achieved complete or partial response. Part of this group (denoted by MAINT in what follows) was subsequently treated with RM, whereas the rest (mainly historical controls, denoted by OBS) remained under observation only. The decision to administer RM depended on the local clinical practice and on the decision of the respective physician.

## 2 METHODS

### 2.1 Study description

Details about the development and operation of the CLSG lymphoma registry have been published elsewhere [2, 17, 18]. Briefly, the CLSG database is a nation‐wide prospectively operated registry collecting data since 1999. It covers approximately 75% of all new lymphoma cases diagnosed in the Czech Republic. This study was a part of the Non‐Hodgkin Lymphoma – Observational Epidemiological and Clinical Study (NiHiL) with ClinicalTrials.gov identifier no. NCT03199066. The study protocol was approved by the independent institutional ethics committee of the General University Hospital in Prague (document no. 1816/15 S‐IV). All patients provided a written informed consent to anonymous processing of their data. All local pathology reports are reviewed by university center‐experienced hematopathologist. In case of inaccurate diagnosis, an additional central pathology review was done centrally by a national lymphoma expert (Vít Campr); if this was not possible, the case was excluded from the analysis.

### 2.2 Patient search

The study comprised patients with grade 1‐3A FL prospectively enrolled in the CLSG network between 1 January 2000 and 31 December 2016 and treated with frontline rituximab‐containing immunochemotherapy. No frontline stem cell (auto/allo) transplant was allowed. All participating centers were called to update data on their patients by the end of January 2020. The database was locked on 15 February 2020.

From a total of 1360 cases identified, 1323 patients were confirmed to have grade 1‐3A FL according to the pathology report review. After preliminary analyses, we decided to include the R‐CHOP induction regimen only to avoid patient selection bias. This is the most frequently used regimen (80%) and the proportion remains stable over time (74.1% and 81.3% in historical OBS and RM cohorts, respectively). Thus, it represents the gold standard for frontline therapy of HTB‐FL in the Czech Republic. Other regimens showed great time‐dependent (fludarabine more used in a historical cohort and bendamustine in the maintenance cohort) or fitness‐dependent (CVP given to older comorbid patients, whereas intensive regimens to younger‐fit ones) variability, leading to great heterogeneity of data.

**FIGURE 1:**
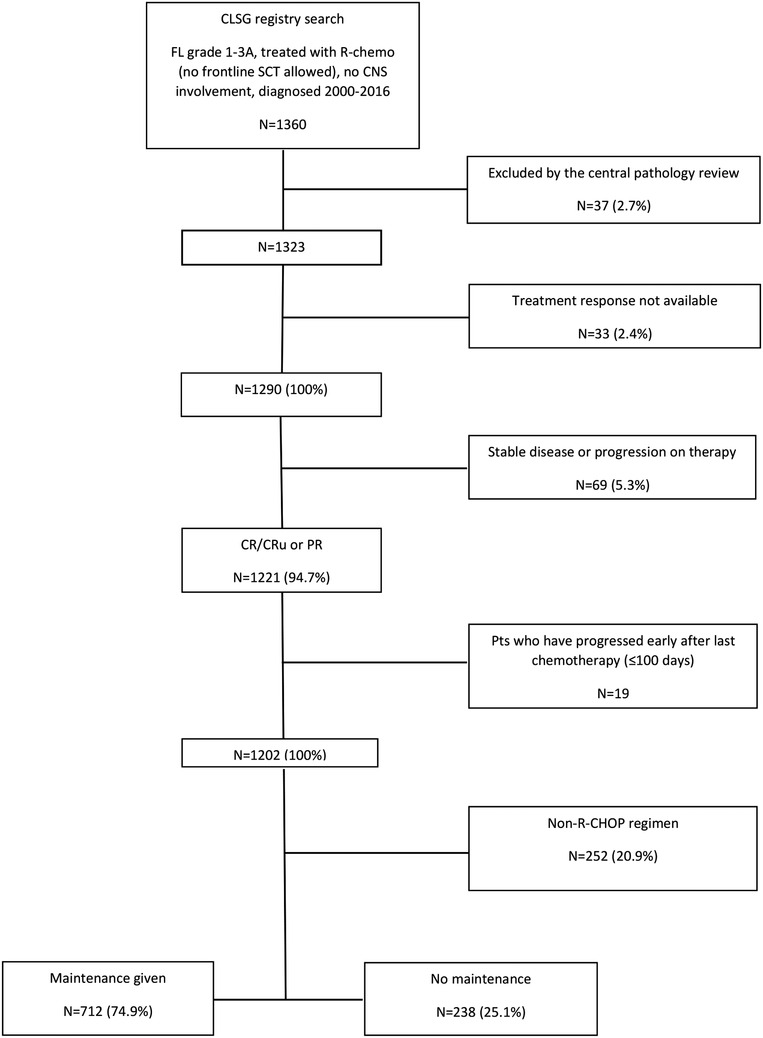
CONSORT flow diagram showing the patient selection process

According to the application of maintenance therapy, patients were allocated to the OBS and MAINT groups. In both groups, patients who relapsed or progressed early were excluded from the analysis to avoid overestimation of the MAINT group performance. This step was meant to exclude those who would have been indicated for RM but had relapsed earlier. Early relapse or progression was defined as an event within 100 days from the date of end‐of‐induction (EOI). The time frame of 100 days was chosen because it was close to the median time (98 days) from the last therapy to maintenance application start. Finally, two cohorts were identified: the OBS group (n = 238) and the MAINT group (n = 712) as shown in the CONSORT flow diagram (Figure 1).

#### 2.2.1 Maintenance schedule

In the MAINT group, patients received RM at a dose of 375 mg/m^2^ every 2 or 3 months, depending on the institutional practice or treating physician's decision [19, 20]. Duration of the maintenance was planned for up to 2 years or until relapse or progression or unacceptable toxicity. The median time from EOI response assessment to RM delivery was 46 days (1.5 months); the median time from the date of the last chemotherapy cycle to the first RM dose was 98 days (3.2 months). The median number of RM doses given was 8; the median duration of RM therapy was 21.3 (range, 1‐33.2) months.

#### 2.2.2 Patient evaluation and response criteria

Treatment response was assessed after frontline therapy. Restaging was done within 2 months (median, 61 days) after administration of the last immunochemotherapy cycle. The response criteria were adapted from the International Workshop to Standardize Response Criteria for Non‐Hodgkin's Lymphomas [21]. Patients were examined by their treating physician every 2 or 3 months during maintenance or at least every 3 months in case of the OBS group.

### 2.3 Statistical analysis

The data were analyzed using the Statistical Package for the Social Sciences (IBM SPSS Statistics for Windows, Version 21.0. Armonk, NY: IBM Corp.). The differences between the OBS and MAINT groups were analyzed using the chi‐squared and Mann‐Whitney *U* tests for qualitative and quantitative variables, respectively. Multivariate analysis was performed using the Cox proportional hazards model fitted to assess the effects of the treatment group and other characteristics on mPOD24. The Kaplan‐Meier method was used to estimate the survival probabilities. The log‐rank test was used to compare difference in survival between patient subgroups. The significance level was set to *P* = .05; two‐tailed tests were used in all the calculations.

Overall survival (OS) was defined as the time from the treatment start (or EOI; OS_EOI_) to the date of the last follow‐up examination (censored) or the date of death (event) from any cause. Progression‐free survival (PFS) was defined as the time from diagnosis (or EOI; PFS_EOI_) to relapse, progression, or death from any cause. Early event (mPOD24) was defined as relapse, progression, transformation, or death from any cause within 24 months after date of the last therapy dose.

To confirm OS benefit of the MAINT group, we randomly pair‐matched OBS group to those receiving RM according to age, sex, and Follicular Lymphoma International Prognostic Index (FLIPI) score (1:1 ratio). The matching procedure was blinded to the outcome in order to avoid selection bias. To confirm appropriate matching, the absence of significant clinical differences between the two cohorts of patients was assessed using Mann‐Whitney *U* test or Fisher's exact test.

OS of the non‐mPOD24 cohort was compared with the age‐, sex‐, and country‐matched general population by the following approach. To each patient, a “digital twin” in the general population of the Czech Republic was defined, who matched the patient in age and gender. This digital twin was followed starting 24 months from EOI of the patient. Mortality tables available at www.mortality.org were used to model the survival of the population of digital twins. The total of 100 digital twin cohorts were modeled and the mean and 5% and 95% quantiles of their survival functions were compared to the survival function of the patient cohort via standardized mortality ratio and expected survival using a conditional approach via the survexp function in R (package survival), modified to allow country of origin as an additional matching feature in a multinational dataset [22]. Population rate tables for countries were obtained via www.mortality.org where available.

## 3 RESULTS

The analyzed cohort consisted of 950 patients who were treated with R‐CHOP, achieved complete or partial response, and did not relapse or progress within 100 days after chemotherapy. Sixty percent of patients were females and the median age at diagnosis was 59 years. The majority of patients (85.5%) had advanced disease stage (Ann Arbor III and IV) and almost half of them (46.4%) had high‐risk FLIPI scores. Table 1 summarizes the population and lymphoma characteristics of the OBS and MAINT groups.

**TABLE 1:** Baseline characteristics of analyzed FL patients from the Czech Lymphoma Study Group registry

Characteristics	Observation	Maintenance	*P*‐value
	N = 238 (100%)	N = 712 (100%)	
Sex, no. male (%)	83 (34.9)	295 (41.4)	.07
Median age, years (range)	58 (26‐78)	59 (26‐83)	.49
Clinical stage (Ann Arbor)			
I	17 (7.1)	21 (3.0)	.03
II	27 (11.3)	70 (9.8)	
III	65 (27.3)	197 (27.7)	
IV	128 (53.8)	424 (59.6)	
N/A	1	0	
Lymphoma grade			
FL 1–2	204 (85.7)	564 (79.2)	.014
FL 3A	33 (13.9)	148 (20.8)	
N/A	1	0	
Bone marrow involvement			
Yes	108 (45.4)	352 (49.4)	.54
N/A			
Beta‐2‐microglobulin			
elevated	93 (39.1)	310 (42.4)	.42
N/A	25 (10.7)	77 (10.8)	
Performance status according to the (ECOG)			
0	119 (50.0)	387 (54.4)	.31
1	104 (43.7)	277 (38.9)	
≥2	12 (5.0)	32 (4.5)	
N/A	2 (0.8)	3 (0.4)	
FLIPI			
Low risk	62 (26.1)	119 (16.7)	.013
Intermediate risk	67 (28.2)	248 (34.8)	
High risk	106 (44.5)	335 (47.1)	
N/A	3 (1.4)	10 (1.4)	
PRIMA‐PI			
Low risk	92 (43.2)	248 (39.1)	.36
Intermediate risk	67 (31.5)	195 (30.7)	
High risk	54 (25.3)	192 (30.2)	
N/A	25 (10.5)	77 (10.8)	
WaW before R‐CHOP			
Yes	15 (6.3)	70 (9.8)	.1
Response after frontline treatment			
CR	162 (68.1)	462 (64.9)	.08
CRu	20 (8.2)	39 (5.5)	
PR	56 (23.5)	211 (29.6)	

Abbreviations: CR, complete remission; CRu, complete remission unconfirmed; ECOG, eastern cooperative oncology group; FL, follicular lymphoma; FLIPI, Follicular Lymphoma International Prognostic index; PR, partial remission, PRIMA‐PI, Primary Rituximab and Maintenance trial‐prognostic index; WaW, watch and wait.

Both patient groups (OBS and MAINT) were well balanced in most variables. There were two notable exceptions. Patients in the MAINT group had higher FLIPI scores (however, the difference in high‐risk FLIPI was less than 3%) and tended to have more advanced disease (stage IV more frequent by 5.8%). The MAINT group had a shorter follow‐up (6.6 vs 11.5 years of the median follow‐up, *P* < .001). The difference in follow‐up was due to historical reasons: most patients treated before 2006 did not receive maintenance therapy because of the absence of reimbursement.

### 3.1 Analysis of treatment response and survival

Overall, the treatment response was known in all 950 patients. Of those, 624 (65.7%) achieved complete (CR), 59 (6.2%) complete‐unconfirmed (CRu), and 267 (28.1%) partial remission (PR). During the follow‐up period, 298 (31.4%) patients progressed or relapsed and 138 (31.4%) died. Five‐year OS (5‐y OS) reached 91.5% (95% CI, 0.89‐0.93) and 5‐year PFS (5‐y PFS) 69.5% (95% CI, 0.66‐0.73) of the entire cohort (not shown).

The histology in relapse was known in 193 cases (64.8%). The transformation to the high‐grade lymphoma was identified in 27 cases (14.0%). Fourteen transformations occurred in the maintenance arm (11.2%), and 13 in the observation arm (19.1%, *P* = .13).

**FIGURE 2:**
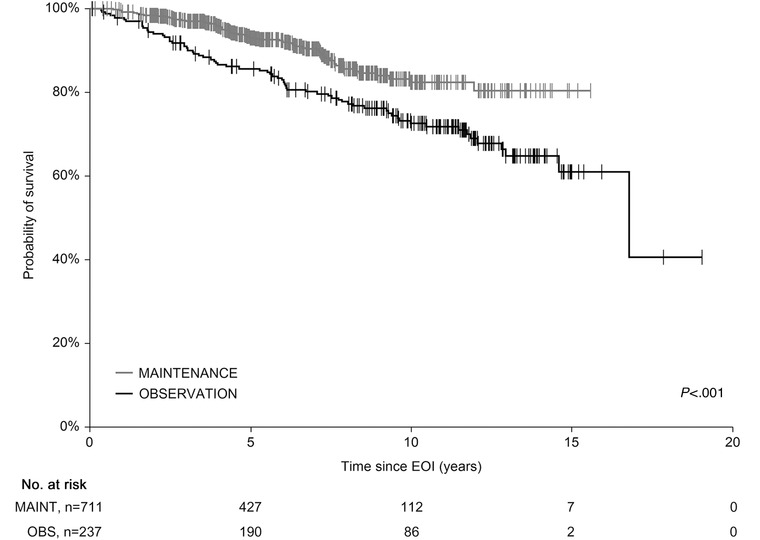
Overall survival (OS) comparison of the observational versus rituximab maintenance cohort

**FIGURE 3:**
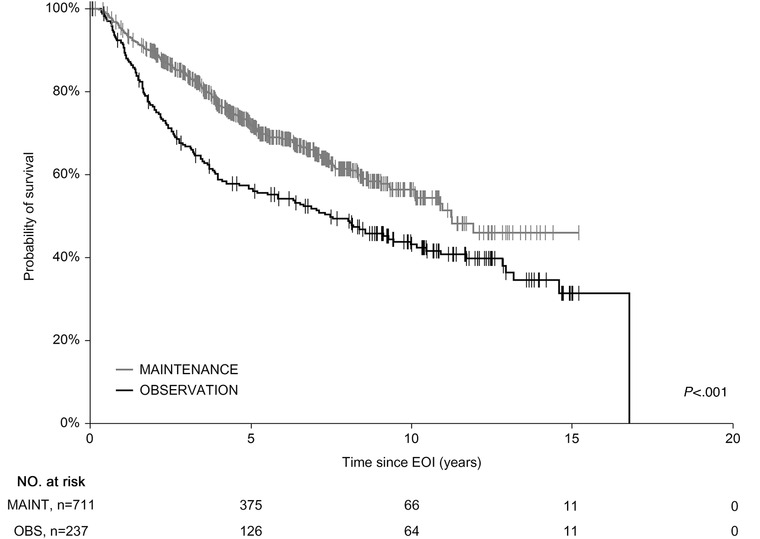
Progression‐free survival (PFS) comparison of the observational versus rituximab maintenance cohort

Subanalysis of the groups showed 5‐y OS_EOI_ of 86.2% and 94.5% in the OBS and MAINT groups, respectively (*P* < .001) (Figure 2) and 5‐y PFS_EOI_ of 58.5% and 75.4%, respectively (*P* < .001) (Figure 3). To confirm OS_EOI_ benefit of the MAINT group, we have paired 235 OBS cases with 235 age‐, sex‐, and FLIPI‐matched MAINT patients. The survival difference was retained with 5‐y OS_EOI_ of 85.7% and 93.7% in the OBS and MAINT groups, respectively (*P* = .007) (not shown).

### 3.2 Analysis of mPOD24 incidence

**FIGURE 4:**
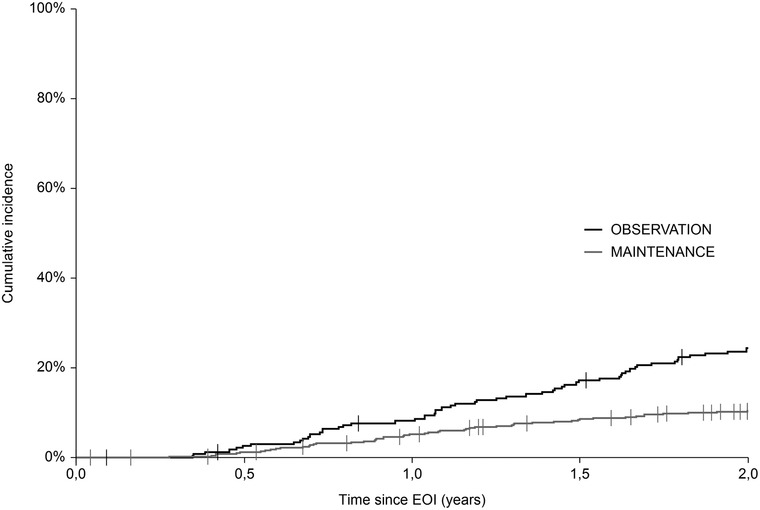
Cumulative incidence curves of mPOD24 events

**FIGURE 5:**
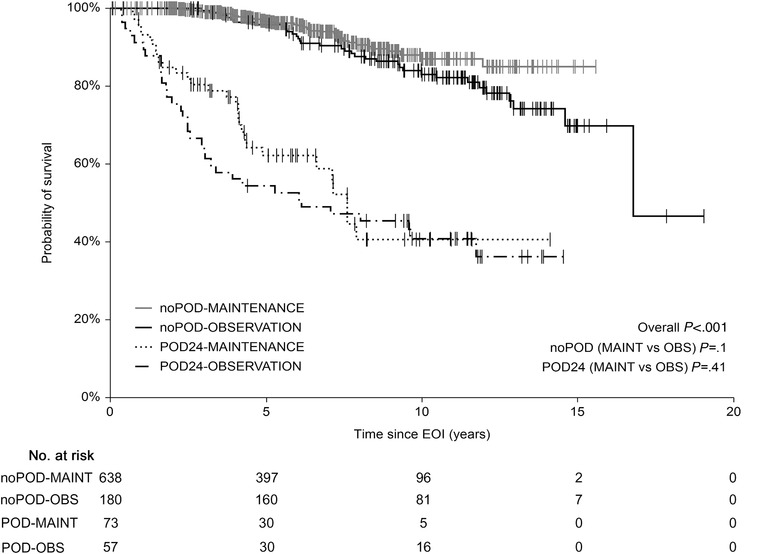
Overall survival since the end of induction (EOI‐OS). Patients stratified according to the mPOD24 incidence and application of rituximab maintenance therapy

A total of 130 events were recorded within 24 months from the EOI (mPOD24), of which 57 (43.8%) were in the OBS group and 73 (56.2%) in the MAINT group. The cumulative incidence of mPOD24 reached 24.1% in the OBS group and 10.1% in the MAINT group (Figure 4; Table 2). Modified POD24 was associated with inferior outcome, regardless of RM application, with 5‐y OS_EOI_ of 49.6% and 62.1% in the OBS and MAINT group, respectively (*P* = .41). On the contrary, all patients without mPOD24 shared outstanding outcome, with 5‐y OS_EOI_ of 95.5% and 96.4% in the OBS and MAINT group, respectively (*P* = .1) (Figure 5).

**TABLE 2:** Description of cumulative incidence of mPOD24 events

In 24 months since EOI:	Observation (n = 238)	Maintenance (n = 712)
POD events, n (%)	57 (23.9)	73 (10.3)
Deaths, n (%)	14 (5.9)	11 (1.5)
2‐year cumulative incidence of mPOD24 events (95% CI)	24.1 (0.19‐0.30)	10.1 (0.08‐0.13)
Relative reduction in risk of mPOD24 events (1 – HR × 100), MAINT vs OBS, % (95% CI)	56.0% (36.0‐56.0)	

Abbreviations: EOI, end of induction; HR, hazard ratio; mPOD24, modified progression of disease within 24 months since EOI; MAINT, maintenance group; OBS, observation group; POD, progression of disease.

**FIGURE 6:**
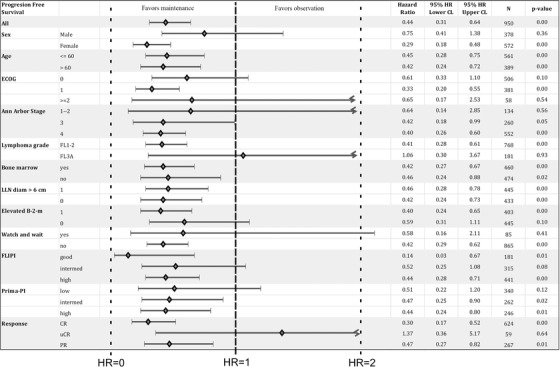
Cox proportional hazards regression analysis showing the effect of rituximab maintenance on the mPOD24 incidence

Exploratory analysis using the Cox proportional hazards model was performed. The only dichotomous predictor variable was assignment to the MAINT group. Figure 6 shows the positive effect of RM on mPOD24 incidence across the population and almost all disease characteristics with overall hazard rate reduction by 56% (hazard ratio = 0.44; *P* < .001). Two exceptions were patients with 3A FL and those who achieved CRu.

**FIGURE 7:**
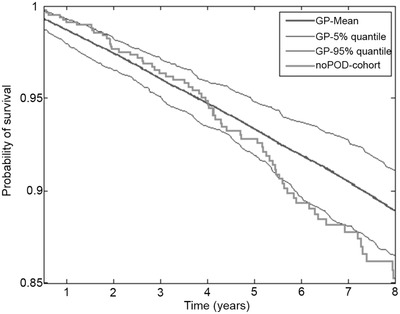
Overall survival of the mPOD24‐free patients compared with general population (GP) survival

The outcome of the mPOD24‐free patients was compared with age‐ and sex‐matched general population data from the Czech Republic population (Figure 7). The data show that mPOD‐24‐free patients share survival within 95% quantile of general population for at least 6 years from the date the mPOD‐24‐free status was achieved. Because the survival is calculated from the date of the end of RM period (or 2 years post EOI in the observation arm), the drop of end of the curve might be influenced by the length of follow‐up. Median follow‐up of the mPOD‐24‐free cohort since EOI is 6.9 years.

## 4 DISCUSSION

FL is still considered incurable. Despite the progress in frontline therapy strategies, lymphoma‐related death remains the most frequent cause of mortality in HTB‐FL patients [23]. It has been clearly shown that the most vulnerable population at risk for lymphoma‐related death is a subgroup of patient relapsing or progressing early after the initial therapy [10]. From this point of view, this event (POD24) should be avoided at all costs.

Maintenance immunotherapy has been established as an optional approach to reducing relapse rates and thus to improve PFS in responding patients with HTB‐FL [4, 5]. Multiple prospective randomized trials have confirmed the benefit of RM in terms of PFS, but not OS compared with observation following frontline immune‐chemotherapy [8, 24]. The OS benefit was not seen even after median follow‐up of 10 years in the PRIMA trial [25]. Effect of maintenance on POD24 events remains poorly studied. Gallium trial comparing rituximab or obinutuzumab in induction and in subsequent maintenance showed fewer early disease progression events occurred in obinutuzumab versus rituximab immunochemotherapy patients, with an average risk reduction of 46.0% and cumulative incidence rate 10.1% versus 17.4% [26]. However, it is unclear, whether POD24 reduction was caused by higher efficacy of particular obinutuzumab‐based induction or due to perhaps more potent obinutuzumab maintenance. Moreover, there are more variables in the game: the obinutuzumab is less effective in individuals with lower drug exposure, in those with low‐affinity Fc receptor variant and with a late NK‐cells recovery [27, 28].

The impact of maintenance with rituximab compared with maintenance naïve population in the context of POD24 has been probably analyzed in the PRIMA trial, but not published yet.

We have done a large landmark analysis of prospectively collected data from the CLSG registry addressing the benefit of RM application. Although the two groups (MAINT and OBS) were not randomized, the assignment of patients to the groups was, to a large extent, dependent on the hospital policy not on their health status. We compared the two groups in terms of survival and analyzed the impact of RM application on mPOD24 defined as an event (progression, relapse, transformation, or death from any cause) within 24 months after date of last chemotherapy dose.

Only chemosensitive patients who achieved CR or PR after the initial R‐CHOP and thus were candidates for RM were included. We found that both the historical (OBS) and MAINT cohorts were well balanced in terms of the vast majority of patient and disease characteristics. There was a trend toward a higher proportion of grade 3A FL, high‐risk FLIPI cases and a slightly lower CR rate in the MAINT group; all these factors are not in favor of the MAINT group and thus do not overestimate the positive effect of RM on survival. We assume that the differences in stage distribution (upstaging) and thus in the FLIPI score, as well as in the quality of response, could be explained by PET/CT being more frequent used in the “modern” MAINT cohort.

The present study confirmed the efficacy of RM application in terms of relapse reduction and PFS benefit but, remarkably, also significant reduction of mPOD24 incidence by almost 60%. This effect was seen across disease and lymphoma characteristics with the exception of grade 3A FL patients and those who achieved CRu. This mPOD24 reduction probably translated into OS benefit of the MAINT group. Survival benefit was confirmed even in the pair‐matched subanalysis with age‐, sex‐, and FLIPI matched maintenance controls. We hypothesize that our 100‐day window after the date of EOI eliminated those ultrahigh‐risk cases in whom RM is not effective [29]. This may also partly explain the absence of the OS benefit in the populations analyzed on the ITT principle [8, 25]. Our observation is consistent with data coming from population registries, confirming OS benefit as well [30, 31]. Interestingly, we have found that once a patient is mPOD24‐free, irrespective of RM application, their survival is comparable to the age‐ and sex‐matched general population.

To conclude, mPOD24 sharply dissects the HTB‐FL population, who responds to initial therapy, into two categories. About one fifth of all patients develop mPOD24, representing the biggest unmet medical need with unsatisfactory response to subsequent treatment and incredibly poor survival. On the other hand, mPOD24‐negative patients enjoy outstanding outcome not much worse than that of the general population in their country. RM therapy might be part of the solution to avoid mPOD24 in a substantial proportion of patients. Given the possible late side effects of RM, we still need more information on who should receive it [29]. Research is underway to offer a more personalized approach with precise staging, biomarker testing, and evolution of novel clinical POD24‐specific prognostic scores [13, 32, 33]. Those efforts are giving us hope that HTB‐FL will be treated in a more personalized way to save lives of those who are at risk and to avoid unnecessary interventions in those who are not [34].

## CONFLICT OF INTERESTS

Vít Procházka provided consultancy (F. Hoffmann‐La Roche AG) and received research funding (Takeda Pharmaceuticals, Inc); Marek Trněný received research funding (Seattle Genetics, Inc), provided consultancy (F. Hoffmann‐La Roche AG), and received speakers´ bureau (F. Hoffmann‐La Roche AG). Other authors declare no conflict of interest.

## DATA AVAILABILITY

The data that support the findings of this study are available on request from the corresponding author. The data are not publicly available due to privacy or ethical restrictions.

## AUTHOR CONTRIBUTIONS

David Belada, Andrea Janíková, and Marek Trněný contributed to the conception and design of the study and wrote the paper. Kateřina Benešová, Heidi Mociková, Juraj Ďuraš, Jan Pirnos, Kateřina Kopečková, Robert Pytlík, Alice Sýkorová, Jozef Michalka, and Tomáš Papajík contributed to the data collection and to follow‐up of the patients. Vít Campr performed pathology supervision. Tomáš Fürst conducted statistical analyses. Jitka Dlouhá was responsible for the data management.
